# Estimations of evapotranspiration in an age sequence of Eucalyptus plantations in subtropical China

**DOI:** 10.1371/journal.pone.0174208

**Published:** 2017-04-11

**Authors:** Wenfei Liu, Jianping Wu, Houbao Fan, Honglang Duan, Qiang Li, Yinghong Yuan, Hao Zhang

**Affiliations:** 1 Jiangxi Provincial Key Laboratory for Restoration of Degraded Ecosystems & Watershed Ecohydrology, Nanchang Institute of Technology, Nanchang, China; 2 Department of Earth, Environmental and Geographic Sciences, University of British Columbia (Okanagan), Kelowna, BC, Canada; Oregon State University, UNITED STATES

## Abstract

Eucalyptus species are widely planted for reforestation in subtropical China. However, the effects of Eucalyptus plantations on the regional water use remain poorly understood. In an age sequence of 2-, 4- and 6-year-old Eucalyptus plantations, the tree water use and soil evaporation were examined by linking model estimations and field observations. Results showed that annual evapotranspiration of each age sequence Eucalyptus plantations was 876.7, 944.1 and 1000.7 mm, respectively, accounting for 49.81%, 53.64% and 56.86% of the annual rainfall. In addition, annual soil evaporations of 2-, 4- and 6-year-old were 318.6, 336.1, and 248.7 mm of the respective Eucalyptus plantations. Our results demonstrated that Eucalyptus plantations would potentially reduce water availability due to high evapotranspiration in subtropical regions. Sustainable management strategies should be implemented to reduce water consumption in Eucalyptus plantations in the context of future climate change scenarios such as drought and warming.

## Introduction

Forests occupy approximately 4.1 billion hectares of the earth’s land surface [1] and play an important role in providing ecological services [2, 3]. In China, forests cover an area of 208 million hectares [4], of which reforested or afforested plantations account for more than one-third of the area. Reforested or afforested plantations have been performing important ecological function and service, which can absorb a large amount of CO_2_ and provide wood products [5, 6]. To meet increasing demands for timber and pulp, fast-growing tree species are widely planted in tropical and subtropical regions to boost regional economy [7, 8]. For instance, Eucalyptus, due to its characteristics of fast-growing, high productivity and good adaptability, has been extensively grown in South China [9]. By 2010, the plantation of Eucalyptus has been expanded to more than ten provinces across China, occupying an area of 3.68 million hectares. Hence, Eucalyptus plantations are of great significance to ecological and economic benefits in South China [9].

In China, studies of Eucalyptus plantations are usually focused on wood production or carbon sequestration [10, 11], nutrients sustainability [12, 13] and native biodiversity [14, 15]. Yet, there is little information on the effects of Eucalyptus plantations on water resources and hydrological processes [16]. Several studies from other countries have demonstrated that expansions of Eucalyptus plantation adversely affect water resources [17, 18]. For example, reduction in streamflow can be attributed to the high water use rates of Eucalyptus for transpiration in coordination with the fast growth [19, 20]. Nevertheless, the relationships between Eucalyptus plantation and water availability still remain inconclusive and thereby deserve further investigations. A better understanding of these relationships can provide insights into the ecohydrological effect of Eucalyptus plantations and support policy making of regional environmental management [21], particularly in South China.

Knowledge of the changes in evapotranspiration of reforested trees across different ages is critical to estimate water use in a forest watershed. Currently, numerous methods have been developed and validated for estimating the transpiration at the individual tree scale and regional scale. For example, the heat pulse velocity (HPV) method was widely used to estimate transpiration rates for Eucalyptus plantations [22, 23]. Several factors, however, constrain the extrapolation of its results at tree scale to a regional scale: a) the number of sampled trees is often small; b) variations in tree growth and unreliable empirical methods can largely contribute to uncertainties for the upscaling [24]; and c) mean sap velocity among individual trees and their stem points are highly variable, and less than four sampling points in each tree are not adequate to explain the range of velocities within the stem [25]. The hydrological models are optimal alternatives to estimate the regional evapotranspiration. Lacking the observational data for model calibration and validation, however, limits it widely application. Therefore, the integration of field observation and hydrological modeling provides a more robust estimation of the effects Eucalyptus plantation on water resources.

Eucalyptus has been introduced to tropical and subtropical regions of China for over 100 years, but studies regarding water use of Eucalyptus is limited [26–28]. The typical rotation time period of planted Eucalyptus in China is about 6–7 years, which enables us to investigate the water use during the rotation period. In addition, the subtropical China is characterized by increased air temperature and seasonal droughts under future climate change [29]. Understanding how water cycles in responses to Eucalyptus plantations are essential to mitigate negative effects of climate change on water resources. Therefore, we aim to (1) estimate the annual evapotranspiration in an age-sequence of Eucalyptus plantations; and (2) to discuss management implications of our results in the context of future climate change.

## Materials and methods

### Study site description

Our study was conducted at the Tianma National Forestry Farm (117° 24' 29'' E, 24° 18' 29'' N), Zhangzhou City, Fujian Province, China. There were no specific permits required for the described field studies. We confirm that the location was not privately-owned or protected in any way, and the field studies did not involve endangered or protected species. Eucalyptus trees have been widely planted for reforestation and cover an area of >0.24 million ha in Fujian Province. (The study was approved by the Ethics Committee of Experimental Center of Tianma National Forestry Farm. Tianma National Forestry Farm issued the permission to conduct this study for each location). This region is characterized by humid subtropical monsoon climate with abundant precipitation. Mean annual temperature is 21°C, with the maximum temperature of 37.7°C in July and the minimum temperature of -1.7°C in January. Mean annual precipitation is 1503 mm, of which 824 mm (47%) and 223 mm (13%) occurs in the wet (April–June) and dry (September–November) seasons, respectively [11].

The main soil type is mountain red soil based on the Chinese soil classification. The soil depth is approximately 100 cm [30, 31]. We selected nine experimental plots with three ages of forest stands on the same soil condition for this study. These Eucalyptus plantations were established after the original vegetation (i.e. Chinese fir forest) was clear removed and then burned under a similar disturbance and topography. In this site, one-year-old *Eucalyptus urophylla×grandis* seedlings were established in 2007, 2009, and 2011, respectively and thus were 2-, 4-, and 6-year-old when this research program was initiated in 2012. The normal rotation period of Eucalyptus plantations in this region is usually 7 years. The selected three ages of Eucalyptus plantations can, therefore, represent the age sequence dynamics of evapotranspiration from planting to harvesting. The seedlings were planted with a spacing of 3 m × 2 m. At the beginning of the establishment, each seedling was fertilized with 500 g of organic fertilizer. Understory species in the nine plantations plots were abundant and dominated by *Pseudosasa amabilis*, *Rubus swinhoei*, *Miscanthus sinensis*, *Dicranopteris dichotoma* and *Smilax china*. The detailed information of site characteristics in the age sequence plantations is shown in Table 1 and also in the reference [11].

**Table 1:** Site characteristics of the different ages of forest plots (Mean ± SE, n = 3).

Plantation age (years)	Previous Forests	DBH (cm)	SOC (g kg^-1^)	TN (g kg^-1^)
2	*Cunninghamia lanceolata*	9.5 ± 0.2	24.9±2.0	2.1±0.04
4	*Cunninghamia lanceolata*	11.7±0.3	18.3±0.8	2.2±0.01
6	*Cunninghamia lanceolata*	13.7±0.3	17.4±0.6	2.0±0.02

DBH, SOC and TN represent as diameter at breast height, soil organic carbon and total nitrogen, respectively.

### Meteorological measurements

Three of nine 20 m × 20 m plots were randomly established for each age class (i.e. three replications). One of three automatic meteorological stations was installed in three replication plots at each age class to monitor precipitation, air temperature, solar radiation, wind speed, soil temperature, and soil water dynamic since December 2013. Automatic meteorological stations located in sites with the open canopy and parted about 9 km among them. Six soil moisture sensors (EM50, USA) were randomly placed in the plot and used to measure soil moisture at 5 cm depth for each age class. Data from January to December 2014 were used for this study.

The historic meteorological data of the study region was obtained from the National Climate Center of China. Two national meteorological stations (Zhangzhou station and Dongshan station) are located approximately 100 km and 80 km away from our study plots, respectively, from which water surface evaporation has been measuring since 1954. In order to improve the accuracy, measured potential evapotranspiration can be obtained by multiplying by a conversion coefficient of 0.733 according to Gong et al.’s method when considering the spatial variation [30].

### Measurement of soil evaporation

From January to December 2014, a simple but effective method, micro-lysimeter (MLS) was used to measure soil evaporation [32–35]. The MLS was made of PVC-material (20 cm in internal diameter and 10 cm in height). The bottom of each MLS was capped with nylon net to allow free drainage of water. Six micro-lysimeters were installed in each plot. The micro-lysimeters were weighed at the same time every day (i.e. 08:00 am) using an electronic balance with a resolution of 0.01 g. For each lysimeter, the difference of weight between 24 hours (one day) is regarded as the daily evaporation from the soil. The soil in MLS was replaced with surrounding soil in every 7 days to avoid any divergence due to the cessation of water exchange with subsoil by root activities [36].

### Estimation of transpiration of Eucalyptus plantations

Monthly potential evapotranspiration (*E*_*0*_) was calculated from monthly temperature by the Hargreaves equation [Eq (1)] [33]. Monthly values are then summed to obtain annual E_0_. Eq (1) is a reliable empirical method and has been widely used to calculate potential evapotranspiration under circumstances of data shortage [34, 35]. The annual actual evapotranspiration (*E*) was calculated using Zhang’s equation [Eq (2)] [37] and Eq (1).

$$E_{0}=0.0023*\;R_{a}\;*\;\left[(T_{\max}+T_{\min})/2+17.8\right]*\;{\left(T_{\max}-T_{\min}\right)}^{0.5}$$ (1)

$$E=P\left[1+w(E_{o}/P)\right]/{\left[1+w(E_{o}/P)+P/Eo\right]}_{}$$ (2)

where, *R*_*a*_ is the extraterrestrial radiation (mm day^-1^); *T*_*max*_ and *T*_*min*_ are the monthly mean maximum and minimum temperature in degree Celsius, respectively; *P* is the precipitation (mm); *E* is the actual evapotranspiration (mm); *E*_*0*_ is the potential evapotranspiration (mm); and *w* is the plant available water coefficient (dimensionless). Given the differences in stand biomass with different plantation ages, the suggested values of vegetation factor *w* for 2-year, 4-year, and 6-year plantations are 0.5, 1 and 2, respectively [37]. This equation was a modification of Budyko’s evaporation by adding an additional vegetation factor *w*, which has been proven to be a sound solution for evapotranspiration estimation at the regional scale [38–41].

It should be noted that evapotranspiration (*E*) in Eucalyptus comes from transpiration from vegetation and evaporation from soil. In this study, rainfall interception was included as vegetation transpiration. Once evaporation from soil was measured, transpiration from vegetation (T_veg_) can then be estimated by the following equation:

$$T_{veg}=E-E_{soil}$$ (3)

### Calculation of Humidity Index (HuI) and relative moisture index (RM)

The average annual Humidity Index (HuI) is the ratio of annual precipitation (*P*) to annual potential evapotranspiration (*E*_*0*_) [42]. The equation is expressed as follow:

$$\text{HuI}=\frac{P}{E_{o}}$$ (4)

The relative moisture index (RM) was recommended as one of drought monitoring factors by China Meteorological Administration in 2005, which indicates soil water balance [43]. The equation is expressed as

$$\text{RM}=\frac{P-E}{E}$$ (5)

where, RM is the relative moisture index (dimensionless), *P* is precipitation (mm), and *E* is evapotranspiration (mm).

### Statistical analysis

One-way ANOVA was conducted to analyze the difference of soil evaporation and soil moisture among age classes. The regression analysis was also performed to test the relationship between soil evaporation and meteorological factors individually. All statistical analyses were conducted with SPSS 13.0 (SPSS, Inc, Chicago, IL). The statistical difference was set at the significance level of 0.05.

## Results

### Monthly dynamics of precipitation and temperature

**Fig 1:**
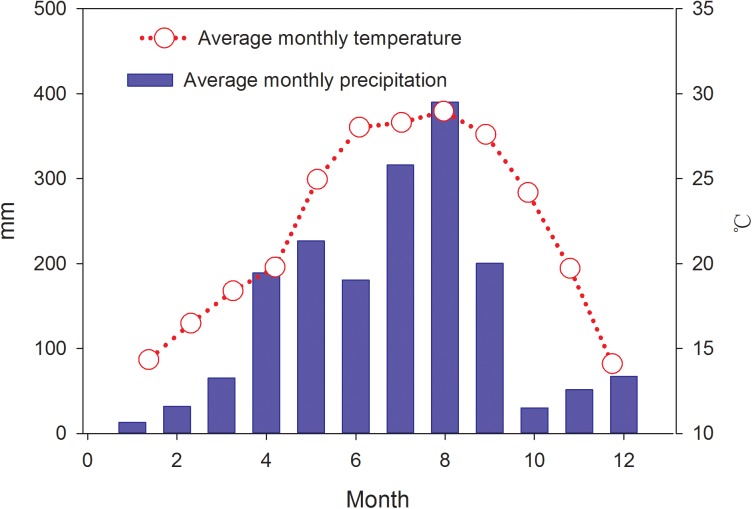
Average monthly precipitation and temperature in the study area for the year 2013.

The monthly precipitation varied throughout the experimental period (Fig 1). The annual total precipitation was 1760 mm, of which 85% occurred from April to September. The average annual temperature was 18.2°C with monthly maximum and minimum temperatures of 35.2°C in July and 1.5°C in January, respectively.

### Monthly dynamics of soil evaporation and soil moisture

**Fig 2:**
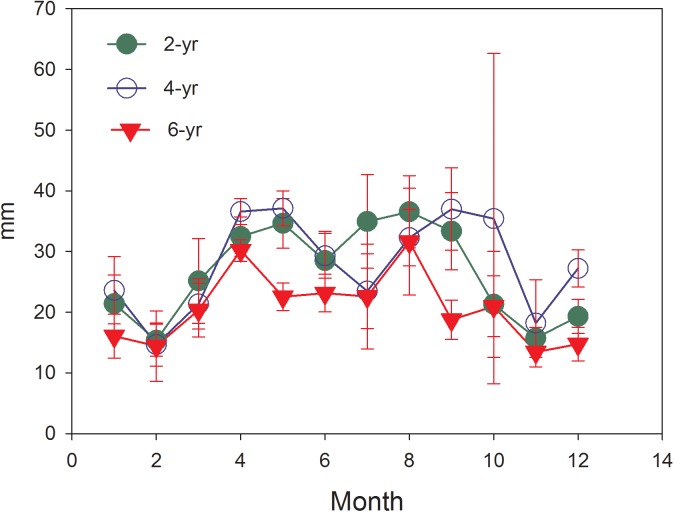
Monthly changes of soil evaporation in different Eucalyptus plantations (the error bar represents the SD).

The estimated annual soil evaporation in the 2, 4 and 6-year-old plantations were 318.6, 336.1, and 248.7 mm, respectively, accounting for 18.1%, 19.1% and 14.1% of annual precipitation. Soil evaporation fluctuated throughout the year (Fig 2). The highest monthly evaporations in 2-year-old plantations (33 mm) and 4-year-old plantations (37 mm) occurred in September, whereas that of 6-year-old plantations (21 mm) shifted to August. The minimum of evaporation occurred in February for 2- (15 mm) and 4-year-old plantations (14 mm), and in November for the 6-year-old plantations (13 mm).

**Fig 3:**
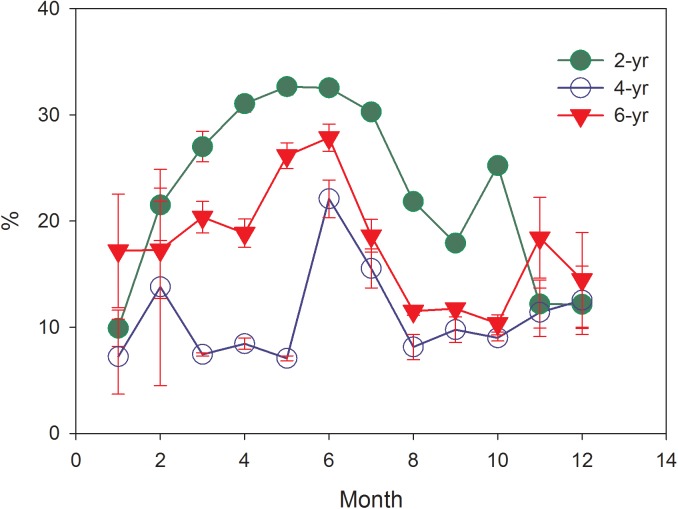
Monthly changes of soil moisture (gravimetric %) in different Eucalyptus plantation forests (at 5 cm depth).

The large seasonal variations of soil moisture (data were collected at 5 cm depth) were found (Fig 3). The annual average soil moisture ranked significantly in the following order: 2-year (22.96%), 6-year (19.10%), and 4-year (12.59%) (*P*<0.05). Soil moisture increased from January to June, while a downward trend was found since June, which was inconsistent with precipitation patterns (Fig 1). Furthermore, in the three plantation plots, no statistically significant relationships were found between soil moisture and precipitation (*P* = 0.089, *P* = 0.124, and *P* = 0.107, respectively).

### Factors affecting soil evaporation

**Fig 4:**
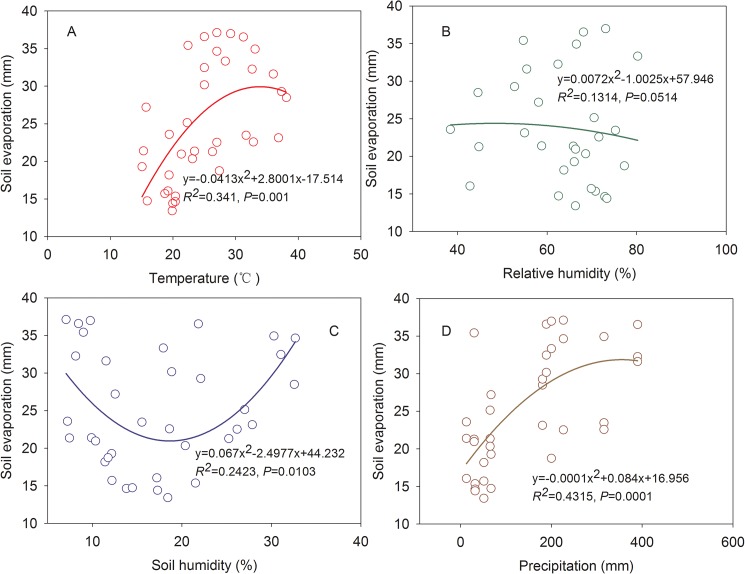
Relationship between soil evaporation and climatic factors i.e. temperature (A), relative humidity (B), soil humidity (C) and precipitation (D).

Regression analyses based on soil evaporation and meteorological factors were conducted to investigate the causality effects of variation in soil evaporation (Fig 4). Results showed that soil evaporation was most sensitive to precipitation (*R*^*2*^ = 0.432, *P*<0.001), followed by the temperature (*R*^*2*^ = 0.341, *P* = 0.001), and soil moisture (*R*^*2*^ = 0.131, *P* = 0.05).

The Humidity Index (HuI) was estimated as 1.35 using Eq (4). Based on Eq (5), the relative moisture index (RM) of 2-, 4-, and 6-year old plantations were 1.01, 0.86, and 1.01, respectively. This indicates that Eucalyptus trees use more water while growing to then slow down the water consumption after maturity [44]; however, due to the limited observations regarding tree ages, we did not detect the critical tree age of water consumption in this study.

### Estimations of transpiration in different Eucalyptus plantations

**Fig 5:**
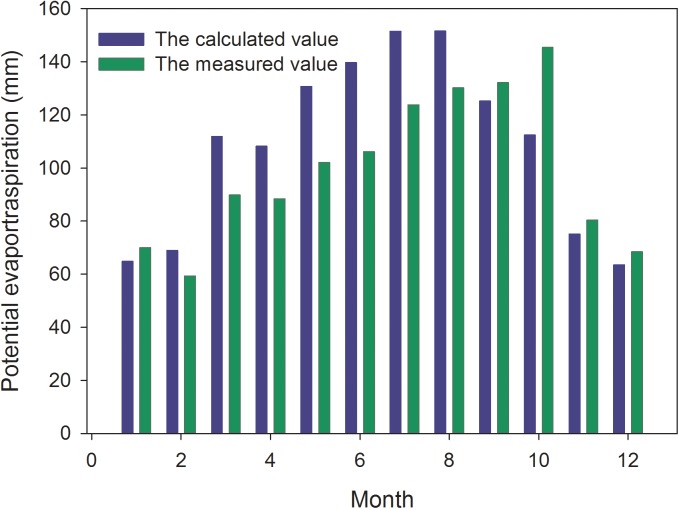
The calculated value of potential evapotranspiration compared with the measured value in the study area. The calculated results based on our plot data by comparing with the measured results based on the historic meteorological data from the National Climate Center of China.

In order to evaluate the accuracy of measured results based on our plot data, our data were compared with the results based on the historic meteorological data from the National Climate Center of China. We compared the difference between the measured and calculated data at a similar climatic condition (Fig 5). In spite of some deviations, the calculated data of empirical formula had relatively high accuracy to represent the *E*_*0*_ of the study area at the annual scale.

**Fig 6:**
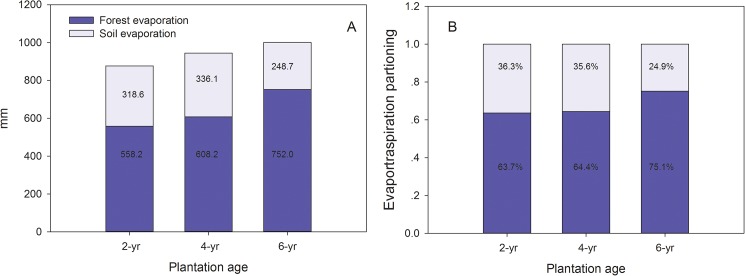
Absolute (A) and relative values (B) of evapotranspiration in different Eucalyptus plantations.

The annual actual evapotranspiration (*E*) calculated by Eq (2). *E* in the 2-year, 4-year, and 6-year Eucalyptus plantations were estimated as 876.8, 944.3, and 1000.7 mm, respectively (Table 2). As mentioned earlier, *E* of Eucalyptus of different ages is composed of soil evaporation and tree transpiration (including understory evaporation and rainfall interception). Vegetation transpiration of 2-, 4-, and 6-year old were respectively estimated as 558.2, 608.2, and 752.0 mm, indicating transpiration increased with increases in tree age (Fig 6). Transpiration from trees of different ages accounted for 31.70%, 34.54%, and 42.70% of precipitation, respectively. The proportion of tree transpiration was elevated along with the increasing plantation ages for *E* of different ages.

**Table 2:** Estimation of evapotranspiration in different Eucalyptus plantations (mm).

plantation age (years)	Month											
	January	February	March	April	May	June	July	August	September	October	November	December
2	12.6	27.9	53.6	89.5	107.9	104.4	131.1	136.7	100.9	28.4	40.2	43.5
4	12.7	28.8	56.0	97.4	117.4	113.0	142.8	148.5	109.7	28.8	42.3	46.7
6	12.8	29.4	57.5	104.1	125.5	119.8	153.1	159.3	117.1	29.1	43.8	49.2

## Discussion

### Tree transpiration along with tree ages

The daily mean transpirations of 2-year, 4-year, and 6-year old trees were estimated as 2.4, 2.6 and 2.7 mm, respectively, by linking model calculations and field experimental measurements. These data were also supported by values obtained using heat dissipation probe method in our study site (i.e. estimated as 2.6, 2.7 and 3.2 mm; unpublished data). It indicated that assignments of the parameter *w* in this study were suitable and accurate to estimate the transpiration of different plantations. It should be noted that the parameter *w* can vary to some extent depending on the differences in tree transpiration among different age classes, which further highlighted a critical need in future studies.

Our findings were consistent with transpiration of Eucalyptus plantations in other countries, ranging from 0.5 to 6 mm·d^-1^ with a higher occurrence in 2–4 mm·d^-1^ [38]. For instance, in the tropical region of China, annual mean daily water use of 4-year-old *Eucalyptus urophylla* plantation was observed as 1.5 mm d^-1^ by the heat dissipation probe method, while transpiration can reach to 4.9 mm·d^-1^ in the wet season [16]. The similar mean daily water use of Eucalyptus plantations (3–8 mm·d^-1^) was also found in the southern India and Brazil [39, 40]. The similar water use was not only found at tree level but also the stand scale [41, 44]. A study conducted in Australia showed that annual mean transpiration was 1000 mm·year^-1^, ranging from 450 mm year^-1^ in the arid area to 1500 mm·year^-1^ in the humid area [41]. In Brazil, tree transpiration varied from 635 mm to 1009 mm and was strongly influenced by variations in annual precipitation and leaf area index [45].

In this study, the increasing trend of total stand transpiration was found from 2-year-old (876.8 mm) to 6-year-old plantations (1000.7 mm). Similarly, a field experiment including six age treatments (2, 4, 5, 6, 7 and 8 years old) was conducted in Victoria, Australia to examine the transpiration of *Eucalyptus globulus* plantations by the heat pulse technique [25]. The results showed that transpiration peaked in the age class of 5–7 years old (i.e. 1.6–1.9 mm day^-1^), while had smaller values in the younger (i.e. 0.4 mm d^-1^ at the age of 2-year-old) or older plantations (i.e. 1.1 mm day^-1^ at the age of 8-year-old). In contrast, there was an opposite trend for older plantation age sequences (> 15 years old) due to typical of saw timber management and thinned stands [25, 46]. For example, a study conducted in the Central Highlands of Victoria, Australia found that annual tree transpiration declined from 733 mm in the 15-year-old forest to 249 mm in the 240-year-old forest [46]. These results generated from studies in longer-rotation forest demonstrated that transpiration increased with increases in age at the early development stage, but declined with age after forest maturity [25, 46].

### Water consumption of Eucalyptus plantations

Our results showed that the soil moisture in the 2-year-old plantations was significantly higher than those of the 4-year and 6-year-old plantations. Previous studies also demonstrated that soil moisture declined with the increase in plantation age, suggesting that the evaporative water loss from soil was in accordance with tree transpiration [47, 48]. The area covered by Eucalyptus plantations has increased, potentially affecting regional water resources. The main reason can be attributed to high transpiration rate of Eucalyptus plantations (including interception losses). Rodríguez-Suarez et al. reported that changes of land cover from fodder maize and pasture to *Eucalyptus globulus* plantation resulted in more rapid declines in the groundwater table [49]. Similarly, Li et al. investigated that groundwater recharge rate across various land uses. The bare land ranks the highest while forest ranks the lowest groundwater recharge rates [50]. The similar field experiment was conducted in Dianbai County, Southern China to study the difference of water table among mixed forest, Eucalyptus plantation and bare land. It indicated that water table depth of mixed forest (ranging from 1 to 4 m below the ground) was lower than the bare land (ranging from 3 to 5 m below the ground). Given the high water consumption of Eucalyptus, water table depth was lowest in the Eucalyptus plantation estimating as from 9 to 11 m [51]. Eucalyptus plantation can be regarded as a major driver to decrease water table [52].

Eucalyptus can consume more water compared to species with slow growing rate due to its high transpiration rates. This is not the unique phenomenon of Eucalyptus compared with other fast growing species. For example, the water use efficiency of Eucalyptus was highest with consumption of water only accounting for 51% and 81% of precipitation compared with conifers and acacia, respectively [16]. In addition, a field experiment focusing on water use of *Pinus radiata* and *Eucalyptus grandis* showed that water use of Eucalyptus was higher than pines, but the difference was not in parallel with the difference in leaf area. When the Eucalypts had a closed canopy, they consumed 22% more water than that of the pines [53]. Therefore, understanding the water consumption of Eucalyptus planations is critical for local water resources.

### Implications for regional forest management

In China, data on demonstrating that Eucalyptus reducing regional water resources is still limited. However, a study conducted in Fuzhou, southeast of China was aimed to analyze dynamics of sap flow between *Eucalyptus spp*. (5-year-old) and Chinese fir (5-year-old) plantations by thermal diffusion and thermal equilibrium method [54]. Results showed that the average daily water consumption of Eucalyptus (1437.4 g d^-1^) was significantly higher than that of Chinese fir (458.12 g d^-1^). In addition, a study comparing the water consumption characteristics between *Eucalyptus urophylla* × *E*. *grandis* seedlings and the local indigenous tree species *Bischofia javanica* in the south China also demonstrated that the average daily water consumption of *Eucalyptus urophylla* × *E*. *grandis* (135.5 g d^-1^) was higher than that of local indigenous tree species (38.1 g d^-1^). These results clearly indicated that Eucalyptus plantations have potential to reduce water availability compared with indigenous tree species [55].

**Fig 7:**
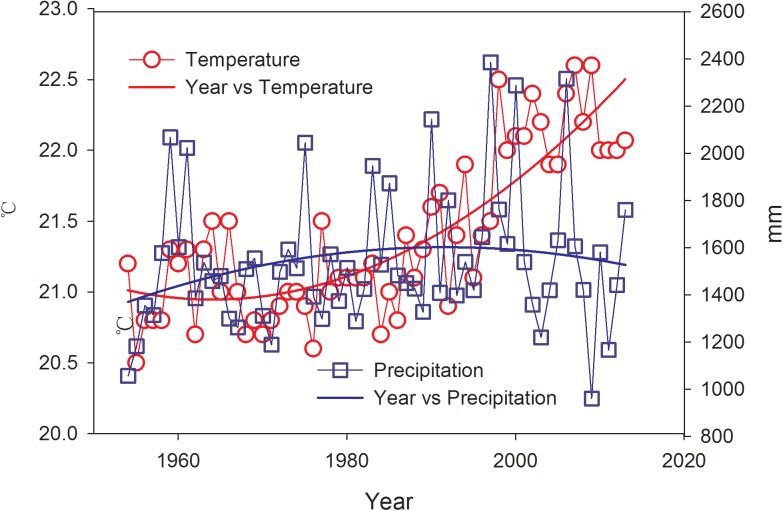
Dynamics of average temperature and precipitation from 1953 to 2013 in the study area.

The humid index (HuI) and the relative moisture index (RM) of our study plots were estimated as 0.35 and 0.88, respectively. In the subtropical region of South China, Yan et al. (2003) analyzed the spatial and temporal variations of hydrological factors in monsoon evergreen broad-leave forest from 1993 to 1999, which showed that RM was 1.01 when precipitation was 1910 mm [56]. However, RM of evergreen broad-leaf forest was 1.08 with a precipitation of 1772 mm in the subtropical region of Southeast China [57]. These results revealed that drought may easily threat Eucalyptus forests compared with broad-leaf forest in a similar precipitation. It is worth mentioning that the temperature of the study area increased significantly (*P*<0.05) from 1954 to 2013, but no significant trends of precipitation were detected (Fig 7). The combined effects would likely to result in an increasing evapotranspiration in near future. IPCC indicates that the precipitation would most likely decrease in the future in our region. The disadvantage of high evapotranspiration will be remarkable and potentially induce water shortages since the water consumption increased with plantation ages of Eucalyptus in our study. Therefore, policy makers should implement a rational distribution for Eucalyptus plantations and shorter rotation periods to minimize the negative hydrological effects in the context of future climate change.

## Conclusions

The efficient evaluation of Eucalyptus evapotranspiration was performed by combining evaporation model and micro-lysimetric measurements in an age sequence of Eucalyptus plantations. First, the total stand transpiration (including interception losses) increased with plantation ages from 2 to 6 years old, which implies the shorter rotation duration (i.e. < 6 years) would diminish the negative effects of Eucalyptus plantations on soil water sustainability. We also found that Eucalyptus plantations may easily suffer drought threat because of their high evapotranspiration. Therefore, policy makers should implement a rational distribution for Eucalyptus plantations to minimize the negative hydrological effects in the context of future climates characterized by rising temperatures and more intense droughts.
